# Effect of inhaled corticosteroid particle size on asthma efficacy and safety outcomes: a systematic literature review and meta-analysis

**DOI:** 10.1186/s12890-016-0348-4

**Published:** 2017-02-07

**Authors:** Céline El Baou, Rachael L. Di Santostefano, Rafael Alfonso-Cristancho, Elizabeth A Suarez, David Stempel, Mark L Everard, Neil Barnes

**Affiliations:** 1 GSK, Middlesex, Stockley Park, Uxbridge, UK; 2 GSK, Research Triangle Park, NC USA; 3 GSK, Upper Providence, PA USA; 4 University of Washington, School of Medicine, Seattle, WA USA; 5 New England Research Institutes, Watertown, MA USA; 6 The University of Western Australia, Crawley, WA 6009 Australia; 7 GSK, Brentford, UK; 8 William Harvey Research Institute, Barts and The London School of Medicine and Dentistry, London, UK; 9 Current affiliation: Janssen Pharmaceuticals, Titusville, NJ USA; 10 Current affiliation: University of North Carolina at Chapel Hill, Chapel Hill, NC USA; 11 PHASTAR, Chiswick, London UK

**Keywords:** Inhaled corticosteroids, Particle size, Asthma, Systematic review, Meta-analysis

## Abstract

**Background:**

Inhaled corticosteroids (ICS) are the primary treatment for persistent asthma. Currently available ICS have differing particle size due to both formulation and propellant, and it has been postulated that this may impact patient outcomes. This structured literature review and meta-analysis compared the effect of small and standard particle size ICS on lung function, symptoms, rescue use (when available) and safety in patients with asthma as assessed in head-to-head randomized controlled trials (RCTs).

**Methods:**

A systematic literature search of MEDLINE was performed to identify RCTs (1998–2014) evaluating standard size (fluticasone propionate-containing medications) versus small particle size ICS medication in adults and children with asthma. Efficacy outcomes included forced expiratory volume in 1 s (FEV_1_), morning peak expiratory flow (PEF), symptom scores, % predicted forced expiratory flow between 25 and 75% of forced vital capacity (FEF_25–75%_)_,_ and rescue medication use. Safety outcomes were also evaluated when available.

**Results:**

Twenty-three independent trials that met the eligibility criteria were identified. Benefit-risk plots did not demonstrate any clinically meaningful differences across the five efficacy endpoints considered and no appreciable differences were noted for most safety endpoints. Meta-analysis results, using a random-effects model, demonstrated no significant difference between standard and small size particle ICS medications in terms of effects on mean change from baseline FEV_1_ (L) (−0.011, 95% confidence interval [CI]: −0.037, 0.014 [*N* = 3524]), morning PEF (L/min) (medium/low doses: −3.874, 95% CI: −10.915, 3.166 [*N* = 1911]; high/high-medium doses: 5.551, 95% CI: −1.948, 13.049 [*N* = 749]) and FEF_25–75% predicted_ (−2.418, 95% CI: −6.400; 1.564 [*N* = 115]).

**Conclusions:**

Based on the available literature, no clinically significant differences in efficacy or safety were observed comparing small and standard particle size ICS medications for the treatment of asthma.

**Trial registration:**

GSK Clinical Study Register No: 202012.

## Background

Asthma is a common chronic lung condition characterized by inflammation of the airways, and defined by episodes of wheezing, chest tightness, shortness of breath, and coughing [1]. Treatment with regular daily inhaled corticosteroids (ICS) is highly effective at reducing symptoms and the risk of asthma exacerbation and is the primary therapy for control of chronic asthma in both adults and children [1]. The clinical effects of daily ICS are recognized in national and international guidelines as they eliminate or reduce chronic symptoms of asthma, prevent exacerbations, maximize lung function, reduce the need for rescue β_2_-agonist treatment, and enable normal activity with few side effects at low and medium dose [1, 2].

Delivery of drug to the lungs is influenced by a number of factors including inspiratory flow and particle size. Current aerosol delivery systems generally deliver poly-dispersed aerosols with the majority of particles in the range 1–5 μm in diameter [3]. Particles <1 μm are generally exhaled while most particles >5 μm are usually deposited in the upper airways. However, altering the characteristics of the aerosol even within this narrow window of 1–5 μm can alter the pattern of deposition within the lungs. As control of asthma by ICS requires delivery to both small and large airways, the differing particle size of ICS medications could potentially impact both efficacy and safety outcomes [4, 5]. Traditional chlorofluorocarbon (CFC) pressurized metered dose inhalers (pMDIs) were all suspension-based formulations but following the CFC transition and the advent of hydrofluoroalkane (HFA) propellants, a variety of new suspension-based and solution-based formulations have been developed. Solution-based pMDIs differ from traditional suspension-based pMDIs in that the respirable particles are only generated after actuation as the propellant evaporates from the liquid plume [6, 7]. The characteristics of the particles generated with solution-based pMDIs vary from formulation to formulation, with some generating extra-fine particles with mass median aerodynamic diameter (MMADs) of <2 μm while others generate particles with MMADs more comparable with traditional HFA-suspension pMDIs (MMADs of 2–5 μm).

Two of the most widely prescribed ICS treatments are fluticasone propionate (FP) and beclometasone dipropionate (BDP), which are chemically and structurally similar but differ in their pharmacodynamic properties [5]. For patients not controlled on ICS alone, both the United States and European guidelines recommend the additional use of a long-acting β_2_-agonist (e.g. salmeterol, formoterol, etc.) in a fixed-dose combination device. FP and FP/salmeterol (FP/SAL) are formulated as HFA-suspensions, while BDP, BDP-formoterol (BDP-F), and a more recent ICS, ciclesonide (CIC) are formulated as HFA-solutions which generate extra-fine aerosols [5]. Thus, FP and FP/SAL are considered standard particle size ICS (2–5 μm), while BDP, BDP-F and CIC are considered small particle ICS (<2 μm).

It has been postulated that the use of ICS medications with a smaller particle size may confer additional clinical benefits to patients with asthma compared with medications with particles of a standard size as they are able to access the smaller airways resulting in increased efficacy [8].

The objective of this systematic literature review and meta-analysis was to evaluate the impact of particle size on clinical outcomes of patients with asthma by comparing the effect of small and standard size particle ICS on lung function, symptoms, rescue use (when available) and safety as assessed in head-to-head randomized controlled trials (RCTs).

## Methods

Details on the methods of the analysis and inclusion criteria were specified in advance and documented in a protocol (GSK Clinical Study Register ID: 202012, data on file), and are summarized below.

### Inclusion criteria, information source, search and study selection

Studies eligible for inclusion in the systematic review were published RCTs comparing FP-containing therapy (standard particle size) with ICS preparations of small particle size in adults and children with asthma. Specifically, treatments evaluated included FP and FP/SAL versus ICS small particle size comparators (BDP, BDP-F or CIC). Abstracts for potential inclusion in the systematic review were identified from the MEDLINE database using the following search terms in PubMed: disease: asthma; exposure: fluticasone, Flovent®, Flixotide®, Advair®, Seretide®. Abstracts in English published between January 1, 1998 and January 13, 2014 were considered.

**Fig. 1:**
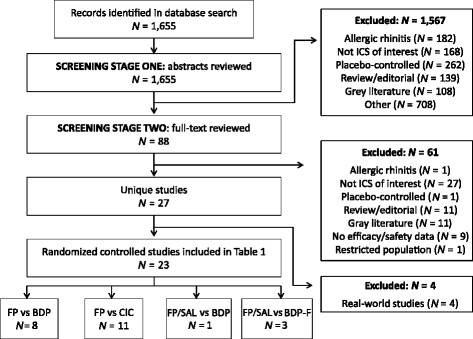
Flow diagram showing implementation of search and screening strategies. BDP, beclometasone dipropionate, BDP-F, beclometasone dipropionate/formoterol fumarate; CIC, ciclesonide; FP, fluticasone propionate; FP/SAL, fluticasone propionate/salmeterol; ICS, inhaled corticosteroid

All identified citations were downloaded and duplicate citations were removed to yield a number of unique hits. Citations were assessed in a multi-stage screening process as outlined in Fig. 1. During Screening Stage One, studies/abstracts were excluded if they included only patients with allergic rhinitis, compared ICS medications other than FP or FP/SAL versus BDP, BDP-F, or CIC, were placebo-controlled, were not a primary epidemiologic/clinical study or were considered ‘gray literature’ (meeting abstracts, letters, websites). Other exclusion criteria included: restricted population (e.g. pregnant women); comparisons of the same ICS at different dosages; no efficacy or safety data. Citations were designated as ‘Exclude’, ‘Include’ or ‘Doubt’ and a record of these decisions was maintained. Abstracts marked as ‘Doubt’ were cross-reviewed by a second epidemiologist. In Screening Stage Two, full text articles of the titles identified as ‘Include’ in Screening Stage One were reviewed and screened against the exclusion criteria listed above. The remaining studies were utilized for extraction.

### Data extraction and data items

Study/patient characteristics and interventions were abstracted from the selected studies. Information on the following efficacy outcome measures were also extracted: forced expiratory volume in 1 s (FEV_1_), morning peak expiratory flow (PEF), asthma symptom scores (on 4–9-point scale where a lower score corresponded to fewer symptoms), % predicted forced expiratory flow between 25 and 75% of forced vital capacity (FVC; FEF_25–75%_)_,_ and rescue medication use per day. In addition to the common lung function measure in asthma studies (FEV_1_ and PEF), FEF_25–75%_ was chosen as an efficacy measure as it is a more sensitive indicator for small airway obstruction than FEV_1_ [9], and thus more likely to demonstrate variations in efficacy if the smaller particles were meeting the small airways. To characterize available safety data, the following endpoints were considered: any adverse events (AEs; at least one), local steroid effects (oral candidiasis, hoarseness), upper respiratory tract infections, growth and bone metabolism, and serum cortisol levels to assess adrenal suppression.

### Assessment of risk of bias

Funnel plots were used to detect biases in the identification and selection of studies. The funnel plot is a technique used to investigate the possibility of biases in the identification and selection phases. In a funnel plot, the estimated effect size of the intervention from individual studies (mean difference) is plotted on the horizontal axis against the standard error of the intervention effect estimate or sample size on the vertical axis. If there are no biases, the graph will tend to have a symmetrical funnel shape centered on the average effect of the studies. All studies were included and additional sources of bias were not formally assessed.

### Planned analysis and statistical methods

Aggregated clinical data from the completed systematic review were summarized in standardized electronic extraction forms, with comparative data also entered into spreadsheets. Clinical statisticians transferred relevant extracted data into SAS (Statistical Analysis Software, Cary, NC) or R (R Foundation for Statistical Computing, Vienna, Austria) for calculation of appropriate statistics and data displays.

The objective of this analysis was to determine if there were any clinically significant differences in the comparative efficacy or safety of FP-containing medications with smaller particle ICS-containing comparators; this was evaluated in the form of a benefit-risk interval plot and/or meta-analysis, when appropriate. The data were extracted on the intent-to-treat (ITT) population as defined in each individual trial. The original publications gave treatment doses as either emitted or delivered; these same doses were reported within this manuscript for consistency.

Treatment comparisons were made using absolute treatment differences between FP-containing formulations and small particle ICS, including 95% confidence intervals (CI). For continuous measures, adjusted mean differences were used, when available. When standard errors (SE) and/or CIs were not directly available, they were estimated using available data [10]. For binary measures, the absolute risk difference and its 95% CI were calculated using the normal approximation to the binomial distribution.

Formal meta-analysis was conducted for efficacy endpoints when there was sufficient sample size and homogeneity across trials. Due to this approach, there was no adjustment for multiple testing. If there was no significant evidence of heterogeneity across the studies, both fixed and random effects models were performed. The statistical heterogeneity of the meta-analysis was assessed by means of the Cochran Q, chi-square test and the I2 statistic with 95% CI. When the assessments such as Cochran Q or chi-square test showed that heterogeneity existed, the results of the random effects model were selected. Results in children (12 years and younger) and adolescents/adults were analyzed separately. Meta regression was used to adjust for differences across studies as appropriate. Additional sensitivity analyses were performed when appropriate.

Where meta-analysis was not feasible, benefit-risk interval plots were produced to visually display the estimated differences between treatments and their 95% CIs for different endpoints on the same graph across studies; irrespective of differences in study designs, endpoints and units.

## Results

The search of the PubMed database identified 1655 potentially relevant articles: 1567 were excluded, mainly because they were placebo-controlled, evaluated allergic rhinitis or did not evaluate an ICS of interest; 88 full-text articles were reviewed and 23 RCTs were included in the final analysis (Fig. 1) [4, 11–32].

Eight studies evaluated FP versus BDP, 11 evaluated FP versus CIC, one evaluated FP/SAL versus BDP and three evaluated FP/SAL versus BDP-F (Table 1). No studies evaluating FP versus BDP-F and FP/SAL versus BDP or CIC were identified. Information for children (6 to 15 years in age) was only available in four studies [15, 18, 26, 27]; one of which utilized a spacer in each arm [18]. No other studies (adults or children) were found to use a spacer.

**Table 1:** Characteristics of RCTs included in the analysis

Reference	Study design and treatment duration	Study population	Treatment group	Comparison group	Efficacy outcome measures (as change from baseline at end of treatment unless otherwise indicated)	Significant results between groups (treatment versus comparison)
Fluticasone propionate (FP) versus beclometasone dipropionate (BDP)						
Aubier, 2001^11^	Open-label, parallel-group 8 weeks	Adults (18–75 years) with ≥4-week clinical history of moderate to severe asthma from 31 medical centers	BDP 800 μg/day via HFA-pMDI (*n* = 101 ITT) High dose	FP 1000 μg via HFA-pMDI (*n* = 97 ITT) High dose	AM PEF (L/min), PM PEF (L/min), FEV_1_ (L), patients free from daily asthma symptoms (%), patients using SABA rescue medication (%)	Equivalence demonstrated for AM PEF: CI within ±25 L/min
Currie, 2002^12^	Single-blind, crossover 3 weeks per Tx period	Patients (mean [SE] age = 38 [4] years) with mild to moderate asthma	FP 500 μg/d via HFA-pMDI (*n* = 20 ITT) Medium dose	BDP 500 μg/d via HFA-pMDI (*n* = 20 ITT) Medium dose	Methacholine PD_20,_ (μg), exhaled tidal NO (ppb), FEV_1_ (% predicted), AM PEF (L/min)	None
			FP 1000 μg/d via HFA-pMDI (*n* = 20 ITT) High dose	BDP 1000 μg/d via HFA-pMDI (*n* = 20 ITT) High dose	Methacholine PD_20,_ (μg), exhaled tidal NO (ppb), FEV_1_ (% predicted), AM PEF (L/min)	None
Fairfax, 2001^13^	Double-blind, double-dummy, parallel-group 6 weeks	Patients 18–65 years with at least a 4-week past history of clinically diagnosed asthma from 30 general practice sites	BDP 400 μg/d via HFA-pMDI (*n* = 88 ITT) Medium dose	FP 400 μg/d via CFC-pMDI (84 ITT) Medium dose	PEFR (L/min), FEV_1_ (L)	Equivalence demonstrated for AM PEFR: CI within ±25 L/min
Ohbayashi, 2008^14^	Double-crossover (results presented from parallel-group analysis of first stage of study) 3 months per Tx	Patients (FP group mean age (years): 61.0 ± 17.4; BDP group mean age: 67.9 ± 13.1) with mild to moderate persistent asthma controlled with FP Diskus for ≤6 months	FP dose dependent on patient, via Diskus (*n* = 25)	BDP Same dose as FP, via HFA-pMDI (*n* = 24)	VC, FVC, FEV_1_, MEFR, PEF (all % predicted), FEF_50%_ (L/s), FEF_75%_ (L/s)	None
Robroeks, 2008^15^	Crossover 3 months per Tx	Children (6–12 years) with moderate persistent asthma from outpatient clinic	BDP 200 μg/d via HFA-pMDI (*n* = 30) Low dose	FP 200 μg/d via DPI (*n* = 30) Low dose	Absolute measures at end of treatment: FEV_1_, FVC, MEF_50_ (all % predicted), FEV_1_/VC (%)	None
Thongngarm, 2005^16^	Open-label 3 months	Patients (≥18 years) with 6-month history of asthma	BDP 320 μg/d via HFA-pMDI (*n* = 20 ITT) Medium dose	FP 330 μg/d via CFC-pMDI (*n* = 10 ITT) Medium dose	FEV_1_, FVC, FEF_25–75%_ (all % predicted), CV (L), CV-VC, post-bronchodilator TLC, TGV, RV, FEV_1_, FVC, FEF_25–75%_ (all % predicted), AM PEF, night-time awakening, shortness of breath, chest tightness, wheezing, cough, phlegm (all symptoms/day), albuterol use (puffs/day)	BDP users had larger improvement in FEF_25–75%_ (% predicted), CV, CV-VC, phlegm and albuterol use
Tunon-de-Lara, 2007^17^	Open, parallel-group 84 days	Patients with mild or moderate asthma	BDP 400 μg/d via HFA-pMDI (*n* = 11 PP) Medium dose	FP 500 μg/d via Diskus (*n* = 14 PP) Medium dose	FEV_1_, FVC, SVC, SVC-FVC, FEV_1_/FVC, FEV_1_/LCV, FEF_50%_ and FEF_25–75%_ (all % predicted)	None
Van Aalderen, 2007^18^	Double-blind, double-dummy, parallel-group 6 weeks	Patients (5–12 years) with asthma dx ≥ 3 months from 6 sites	BDP 200 μg/d via HFA-pMDI (*n* = 139 ITT) Low dose	FP 200 μg/d via CFC-pMDI (*n* = 140 ITT) Low dose	AM PEF, PM PEF, FEV_1_, FVC, FEF_25–75%_ (all % predicted), symptom-free days (%), nights without sleep disturbance (%), β_2_-agonist therapy,mean (puffs/day)	Noninferior: lower CL > −5 (% predicted) for AM PEF; FP users had greater improvement in FEF_25–75%_ (% predicted)
Fluticasone propionate versus extra-fine ciclesonide (CIC)						
Bateman, 2008^19^	Open-label, parallel-group 24 weeks	Outpatient adults and adolescents (12–75 years) with asthma in Europe	CIC 640 μg/d via HFA-pMDI (*n* = 255 ITT) High dose	FP 660 μg/d via HFA-pMDI (*n* = 273 ITT) High dose	FEV_1_ (mL), FVC (L), PEF (L/min), AM PEF (L/min), asthma symptom score, rescue medication use (puffs/day) Absolute measures at end of treatment: Asthma symptom-free days, rescue medication-free days, asthma symptom and rescue medication-free days	Noninferior: lower CL above −200 mL for FEV_1_; Noninferior: lower CL above −0.2 L for FVC; Noninferior: lower CL above -25 L/min for PEF and AM PEF
Boulet, 2007^20^	Open-label, parallel-group 12 weeks	Adult and adolescent (12–75 years) patients with asthma from 59 Tx centers	CIC 320 μg/d via MDI (*n* = 233 ITT) Medium dose	FP 400 μg/d via DPI (*n* = 239 ITT) Medium dose	FEV_1_ (mL), FEV_1_ (% predicted), FVC (L), SVC (L), AM PEF (L/min), PM PEF (L/min), daytime asthma symptom score, total asthma symptom score, rescue medication use (puffs/day)	Noninferior: lower CL above −200 mL for FEV_1_; Noninferior: lower CL above −0.2 L for FVC; Noninferior: lower CL above −25 L/min for AM PEF and PM PEF
Buhl, 2006^21^	Multicenter, double-blind, double-dummy, parallel-group 12 weeks	Adult and adolescent (12–75 years) patients with asthma from 57 Tx centers	CIC 160 μg/d (*n* = 266 ITT) Low dose	FP 176 μg/d via HFA-pMDI (*n* = 263 ITT) Low dose	FEV_1_ (L), FVC (L), AM PEF (L/min), daytime symptom score, night-time symptom score, total asthma symptom score (day + night score), rescue medication use (puffs/day)	Noninferior: lower CL above -0.2 L for FEV_1_ and FVC; Noninferior: lower CL above -25 L/min for AM PEF
Cohen, 2011^4^	Double-blind, double-dummy, parallel-group 65 days	Adult patients with asthma (18–60 years) recruited from outpatient clinics of pulmonology departments in the Netherlands	CIC 160 μg/d via HFA-pMDI (*n* = 19) Low dose	FP 200 μg/d via HFA-pMDI (*n* = 18) Low dose	FEV_1_ (% predicted), FVC (L), SVC (L), FVC/SVC (L), FEF_50%_ (L/s), FEF_25–75%_ (% predicted)	FP users had greater improvement in FEV_1_ and FEF_25–75%_ (% predicted)
Dahl, 2010^22^	Double-blind, double-dummy, 2-arm, parallel-group 24 weeks	Adult and adolescent patients (12–75 years) with mild to moderate asthma from 48 centers internationally	CIC 80 μg/d via HFA-pMDI (*n* = 240 ITT) Low dose	FP 200 μg/d via HFA-pMDI (*n* = 240 ITT) Low dose	FEV_1_ (L), FVC (L), AM PEF (L/min) Absolute measures at end of treatment: Days with asthma control (%), asthma exacerbations requiring Tx (%)	Noninferior: lower CL above -0.2 L for FEV_1_ and FVC; Noninferior: lower CL above -25 L/min for AM PEF
Lee, 2004^23^	Double-blind, double-dummy, crossover 4 weeks per Tx	Non-smoking patients with mild-to-moderate persistent asthma	CIC 400 μg/d via HFA-pMDI (*n* = 19) High dose	FP 500 μg/d via HFA-pMDI (*n* = 19) Medium dose	NO (ppb), FEV_1_ (L), FEV_1_ (% predicted), FEF_25–75%_ (L/s), FEF_25–75%_ (% predicted), methacholine PD_20_ (mg/ml), AM PEF (L/min), PM PEF (L/min), NO (ppb), AM and PM asthma symptom score, AM and PM rescue medication use (puffs/day)	None
Lee, 2005^24^	Double-blind, double-dummy, crossover 4 weeks per Tx	Adult patients (mean age 47 years) with moderate, persistent asthma stable for 3 months	CIC 1600 μg/d via HFA-pMDI (*n* = 14) High dose	FP 2000 μg/d via HFA-pMDI (*n* = 14) High dose	Absolute measures at end of treatment: FEV_1_ (L), FEV_1_ (% predicted), FEF_25–75%_ (L/s), FEF_25–75%_ (% predicted), AM PEF (L/min), PM PEF (L/min), AM and PM asthma symptom score, AM and PM rescue meds (puffs/d), exhaled nitric oxide (ppb), methacholine PC_20_ (mg/mL)	None
Magnussen, 2007^25^	Double-blind, double-dummy, 3-arm, parallel-group 12 weeks	Patients at least 12 years of age with asthma from 91 sites	CIC 80 μg/d via HFA-pMDI (*n* = 278 ITT) Low dose	FP 178 μg/d via HFA-pMDI (*n* = 259 ITT) Low dose	FEV_1_ (mL), FVC (L), PEF (L/min), asthma symptom score, daytime and night-time symptom score, rescue medication use (puffs/day)	Noninferior: lower CL above -200 mL for FEV_1_; Noninferior: limit NR for FVC and PEF
			CIC 160 μg/d via HFA-pMDI (*n* = 270 ITT) Low dose	FP 178 μg/d via HFA-pMDI (*n* = 259 ITT) Low dose	FEV_1_ (mL), FVC (L), PEF (L/min), asthma symptom score, daytime and night-time symptom score, rescue medication use (puffs/day)	Noninferior: lower CL above -200 mL for FEV_1_; Noninferior: limit NR for FVC and PEF
Pedersen, 2006^26^	Multicenter, double-blind, double-dummy, 2-arm, parallel-group 12 weeks	Children (6–15 years) with asthma from 51 sites	CIC 160 μg/d via HFA-pMDI (*n* = 254 ITT) Low dose	FP 178 μg/d via HFA-pMDI (*n* = 257 ITT) Low dose	FEV_1_ (L), PEF (L/min), AM PEF (L/min), PM PEF (L/min), asthma symptom score sum, asthma symptom free days, rescue medication-free days	Noninferior: lower CL greater than −0.1 L for FEV_1_; Not noninferior: lower CL less than −12.5 L/min for PEF, AM PEF and PM PEF
Pedersen, 2009^27^	Double-blind, double-dummy, 3-arm, parallel-group 12 weeks	Children (6–11 years) with asthma from 50 international centers	CIC 80 μg/d via HFA-pMDI (*n* = 234 ITT) Low dose	FP 178 μg/d via HFA-pMDI (*n* = 245 ITT) Low dose	FEV_1_ (L), AM PEF (L/min), asthma symptom score sum, rescue medication use Absolute measures at end of treatment: Asthma exacerbations (%)	Noninferior: lower CL greater than −0.1 L for FEV_1_; Not noninferior: lower CL less than −12.5 L/min for AM PEF; Noninferior: upper CL less than 0.30 for asthma symptom score sum
			CIC 160 μg/d via HFA-pMDI (*n* = 232 ITT) Low dose	FP 178 μg/d via HFA-pMDI (*n* = 245 ITT) Low dose		Noninferior: lower CL greater than −0.1 L for FEV_1_; Noninferior: lower CL greater than −12.5 L/min for AM PEF; Noninferior: upper CL less than 0.30 for asthma symptom score sum
Van der Molen, 2010^28^	Multicenter, parallel-group (series of 3 studies) 12–24 weeks	Health patients with asthma? aged 12–75 years	CIC 320 μg/d via pMDI (*n* = 224 ITT) Medium dose	FP 400 μg/d via DPI (*n* = 228 ITT) Medium dose	No efficacy outcomes; only safety outcomes available	
			CIC 640 μg/d via pMDI (*n* = 244 ITT) High dose	FP 750 μg/d via pMDI (*n* = 254 ITT) High dose		
			CIC 640 μg/d via pMDI (*n* = 250 ITT) High dose	FP 1000 μg/d via pMDI (*n* = 237 ITT) High dose		
Fluticasone propionate/salmeterol versus extra-fine beclometasone dipropionate						
Fowler, 2002^29^	Double-blind, double-dummy, parallel-group 8 weeks	Patients with asthma aged 16–70 years	BDP 400 μg/d via HFA-pMDI (*n* = 20) Medium dose	FP/SAL 200/100 μg/d via DPI (*n* = 19) Low dose	Absolute measures at end of treatment: Methacholine PD_20_ (μg), FEV_1_ (L), FEV_1_ (% predicted), FEF_25–75%_ (L/s), FEF_25–75%_ (% predicted), AM PEF (L/min), PM PEF (L/min), tidal exhaled NO (ppb), symptom score, reliever use (puffs/d)	FP/SAL users had higher post-study methacholine PD_20_, FEV_1_ (L), FEV_1_ (% predicted), post-study AM/PM PEF
Fluticasone propionate/salmeterol versus extra-fine beclometasone dipropionate/formoterol fumarate						
Papi, 2007^30^	Double-blind, 2-arm parallel-group 12 weeks	Patients with asthma aged 18–65 years from 12 outpatient respiratory clinics in Europe	BDP-F 400/24 μg/d via pMDI (*n* = 115 ITT) Medium dose	FP/SAL 500/100 μg/d via pMDI (*n* = 113 ITT) Medium dose	AM pre-dose PEF, AM PEF and PM PEF (all L/min), FEV_1_ (L), FVC (L), Absolute measures at end of treatment: Daytime and night-time symptom score, symptom-free days (%), day time use of rescue medication (puffs)	Noninferior: lower CL above -20 L/min for AM pre-dose PEF; BDP-F users had greater improvement in FVC
Papi, 2012^31^	Prospective, controlled, 2-arm parallel-group 24 weeks	Patients with asthma aged 18–65 years from 67 respiratory clinics in Europe	BDP-F 400/24 μg/d via pMDI (*n* = 206 ITT) Medium dose	FP/SAL 500/100 μg/d via Diskus DPI (*n* = 216 ITT) Medium dose	Absolute measures at end of treatment: AM PEF (L/min), FEV_1_ (L), FEV_1_ (% predicted), PEF (L/min), PEF (% predicted), daytime and night-time symptom score, symptom-free days (%), patients with controlled asthma, asthma exacerbations and severe asthma exacerbations (all %)	Equivalent: CI falls within − 20 to +20 L/min for AM PEF
Scichilone, 2010^32^	Double-blind, double-dummy, parallel-group 12 weeks	Adult patients with asthma (18–50 years) in Italy	BDP-F 400/24 μg/d via HFA-pMDI (*n* = 15) Medium dose	FCS 500/100 μg/d via pMDI (*n* = 15) Medium dose	Absolute measures at end of treatment: Pre-dose FEV_1_ (L), FEF_25–75%_ (L/s), PD_20_ FEV_1_ methacholine (μg)	None

*AM*, morning; *CFC*, chlorofluorocarbon; *Cl*, confidence interval; *CL*, clearance; *CV*-*VC*, closing volume/vital capacity; *DPI*, dry powder inhaler; *dx*, diagnosis; *ER*, emergency room; *FEF*, forced expiratory flow; *FEV*_1_, forced expiratory volume in 1 sec; *FVC*, forced vital capacity; *HFA*-*pMDI*, hydrofluoroalkane-pressurized metered dose inhaler; *ITT*, intent-to-treat; *NO*, nitric oxide; *NR*, not reached; *PEFR*, peak expiratory flow rate; *PM*, evening; *PP*, per protocol; ppb, part per billion; *RV*, residual volume; *SABA*, short-acting beta agonists; *SVC*, slow vital capacity; *TGV*, thoracic gas volume; *TLC*, total lung capacity; *Tx*, treatment

The main efficacy endpoints evaluated in the studies were FEV_1_ and PEF (Table 1). The predominant safety endpoints were overall incidence of AEs and urinary cortisol levels.

### Fluticasone propionate versus beclometasone dipropionate

In the eight identified trials comparing conventional suspension-based FP pMDIs with the ‘ultra fine’ solution-based BDP formulations similar doses have been used in each arm (Table 1). This is in accordance with GINA guidelines reporting the clinically comparable doses of HFA-FP and HFA-BDP [1]. Hence these comparisons will address the issue of whether differences in particle size results in a change in the efficacy or safety profile.

The majority of the RCTs reported no significant difference in efficacy outcome measures between FP and BDP. Two of the eight RCTs reported significant differences in FEF_25–75%_ between FP and BDP; one demonstrating improvement with FP [18] and the other with BDP [16] (Table 1).

The majority of RCTs reported no significant difference in AEs or other safety markers between the two treatments. Overnight urinary cortisol/creatinine production was suppressed more in patients using BDP compared with patients on FP (1000 μg/day FP versus BDP: geometric mean fold difference 1.97 [95% CI: 1.28, 3.02]; *p* < 0.05) [12]. No significant difference was found in cortisol levels in three studies, either in levels at the end of the treatment period or in change from baseline [11, 13, 14].

### Fluticasone propionate versus ciclesonide

With few exceptions in 10 identified RCTs, CIC was found to be non-inferior or not statistically different from FP for numerous efficacy endpoints (Table 1). Notably, in a trial by Pedersen et al. in children aged 6–11 years, non-inferiority of CIC to FP (88 μg twice daily) was observed with regard to change in FEV_1_ from baseline over 12 weeks of treatment with 160 μg but not 80 μg once daily [27]. A similar trial in children aged 6–15 years comparing the same doses found that CIC did not show non-inferiority in the change from baseline in morning or afternoon PEF following 12 weeks of treatment [26]. One trial of adult patients, by Cohen et al., found greater improvement in lung function among patients receiving FP compared with patients receiving CIC, specifically in mean ± SD % predicted FEV_1_ (0.5 ± 4.3 versus −3.0 ± 4.6, respectively; *p* = 0.021) and FEF_25–75%_ (0.6 ± 5.6 versus −3.6 ± 6.0, respectively; *p* = 0.034) [4].

Results for differences in urinary cortisol levels (adjusted for creatinine) were variable, with greater adrenal suppression among patients receiving FP (compared with CIC) reported in an ITT analysis restricted to patients with normal creatinine levels (*p* = 0.006) [26]. In a small crossover study, patients on FP 2000 μg/day had significantly lower mean overnight 10-h urinary cortisol than patients on CIC (CIC versus FP: geometric mean fold difference 1.5 [95% CI: 1.1, 2.0]; *p* < 0.05) [24]. One trial assessed side effect perception using a 100-point scale, and observed that patients on CIC had either a smaller increase in perceived side effects or a decrease over the treatment period from baseline compared with patients receiving FP (between-treatment least squares mean (±SE) in total Inhaled Corticosteroid Questionnaire Scores: CIC 320 μg once-daily versus FP 200 μg twice-daily (12 weeks): −2.52 ± 0.82, *p* = 0.0011; CIC 320 μg twice-daily versus FP 500 μg twice-daily (24 weeks): −2.05 ± 0.79, *p* = 0.0047 [28].

### Fluticasone propionate/salmeterol versus beclometasone dipropionate

One trial was identified that compared the efficacy of BDP (400 μg/day) and FP/SAL (200 μg/100 μg/day) as a step-down therapy after high-dose ICS (dry powder inhaler [DPI]-BDP 2000 μg/day) (Table 1) [29]. Lung function measures were compared between treatment groups at the end of the 8-week treatment period instead of comparing the change from baseline in each group. Methacholine PD_20_, post-study FEV_1_ (measured and % of predicted), morning and afternoon PEF were all significantly greater in patients on FP/SAL than in patients on BDP (methacholine PD_20_ (μg): 149.9 [95% CI: 114.3, 196.5] versus 71.2 (95% CI: 54.7, 92.8]; FEV_1_ (L): 2.46 [95% CI: 2.39, 2.53] versus 2.26 [95% CI: 2.20, 2.33], *p* < 0.05; FEV_1_ (% predicted): 77 [95% CI: 75, 79] versus 70 (95% CI: 68, 72], *p* < 0.05; morning PEF (L/min): 434 [95% CI: 424, 445] versus 402 [95% CI: 391, 411], *p* < 0.05; evening PEF (L/min): 436 [95% CI: 425, 446] versus 408 [95% CI: 398, 418]; *p* < 0.05). No differences were found in FEF_25–75%_, symptom scores, or reliever medication use [29]. No significant differences were found between treatment groups for serum cortisol levels, urinary cortisol/creatinine ratio, or serum osteocalcin [29].

### Fluticasone propionate/salmeterol versus beclometasone dipropionate-formoterol

Three RCTs compared the efficacy of FP/SAL with BDP-F (Table 1) [30–32]. One trial in adults with asthma found significantly greater improvement in FVC in patients receiving BDP-F than in those receiving FP/SAL (0.46 ± 0.51 L versus 0.34 ± 0.44 L, respectively; *p* = 0.040); however, no differences were found for any other efficacy parameters [30].

Two RCTs were identified that compared the safety of BDP-F with FP/SAL; no differences in AEs or urinary cortisol/creatinine ratio were observed between the two treatments [30, 31].

#### Meta-analysis

**Fig. 2:**
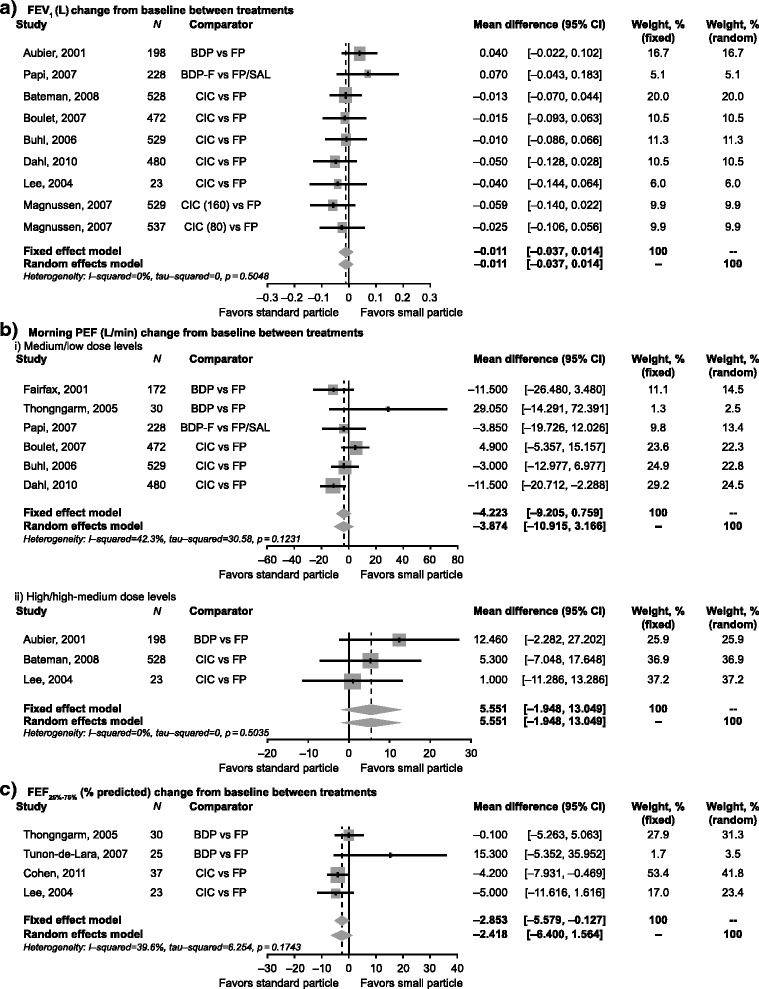
Pooled effects for efficacy endpoints with 95% CI of eligible studies comparing small versus standard size particle ICS medications. Mean difference in change from baseline between treatments for (**a**) FEV_1_, (**b**) morning PEF and (**c**) FEF_25%-75%._ BDP, beclometasone dipropionate, BDP-F, beclometasone dipropionate/formoterol fumarate; CI, confidence interval; CIC, ciclesonide; FEF_25–75%,_ % predicted forced expiratory flow between 25% and 75% of forced vital capacity; FEV_1_, forced expiratory volume in 1 s; FP, fluticasone propionate; FP/SAL, fluticasone propionate/salmeterol; PEF, peak expiratory flow

Meta-analysis methods could only be applied for the efficacy endpoints of FEV_1_, PEF, and FEF_25–75%_ (Fig. 2). Other efficacy and safety endpoints were not considered for the meta-analysis due to heterogeneity, potential publication bias, and disparity of endpoint definitions and/or timing of collection. In adults, the random effects models showed no significant differences between small and standard size particle ICS for change in FEV_1_ (−0.011 L, 95% CI: −0.037, 0.014; *p* = 0.394), or FEF_25–75%_ (−2.418, 95% CI: −6.400, 1.564; *p* = 0.234) (Figs. 2a and c).

Meta-regression analysis showed that the only treatment effect modifier present for morning PEF was dose level (high versus low); however, high dose versus medium dose or high hose versus high-medium dose did not show a statistically significant difference. This suggested that it may not be appropriate to use either meta-regression or meta-analysis with all of the data in the final model. Instead, the morning PEF endpoint was analyzed as two separate subgroups (high/high-medium doses and medium/low doses). The random effects models showed no significant differences between small and standard size particle ICS for change in morning PEF (medium/low doses: −3.874 L/min, 95% CI: −10.915, 3.166; high/high-medium doses: 5.551 L/min, 95% CI: −1.948, 13.049) (Figs. 2bi and ii).

For each endpoint, the heterogeneity test showed that there was no between-study variation, suggesting that fixed effects models were also appropriate. The random effects models, which provide a more conservative approach, were retained as primary models. Similar to the random effects model, no significant differences were observed for change in FEV_1_ and morning PEF using a fixed effects model (*p* = 0.394 and *p* = 0.097, respectively). Even though the analysis of both data subgroups for the morning PEF data led to the same conclusion, the results for the mean differences between medium/low (−4.223 L/min) and high/high-medium (5.551 L/min) dose levels appeared to be in opposite directions (Figs. 2bi and ii). Treatment differences in FEF_25–75%_ were found to be significantly in favor of FP using a fixed effects model (−2.853 L/min; 95% CI −5.579, −0.127; *p* = 0.040), though not in the random effects model (−2.418, 95% CI: −6.400, 1.564; *p* = 0.234) (Fig. 2c). The heterogeneity test and I^2^ (*p*-value = 0.174 and I^2^ = 39.6%) showed no significant between-study-variation. However, definitive conclusions could not be drawn from these treatment differences due to the small number of studies (*N* = 4) evaluated, which also explains the wider CIs for the results of the random effects model. In children, the small number of studies with disparate endpoints and results did not allow for meta-analysis.

Sensitivity analyses were performed for FEV_1_, the high/high-medium dose subgroup data for morning PEF, and FEF_25–75%_, by excluding trials with crossover design [23] or with multiple arms (only in the case of FEV_1_) [25]. The results of the sensitivity analyses for FEV_1_ and the morning PEF were similar to the results of the final model. However, the results of the sensitivity analysis for FEF_25–75%_ (excluding Lee et al. [22]), were found to differ from the final model. There was a statistically significant treatment difference between standard size and small size particles on FEF_25–75%_ in the final fixed effect model (−2.853 L/min; 95% CI −5.579, −0.127; *p* = 0.040) but not in the sensitivity analysis (−2.414 L/min; 95% CI −5.406, 0.578; *p* = 0.114). These conflicting conclusions may be due to small patient sample sizes.

#### Benefit-risk plots

**Fig. 3:**
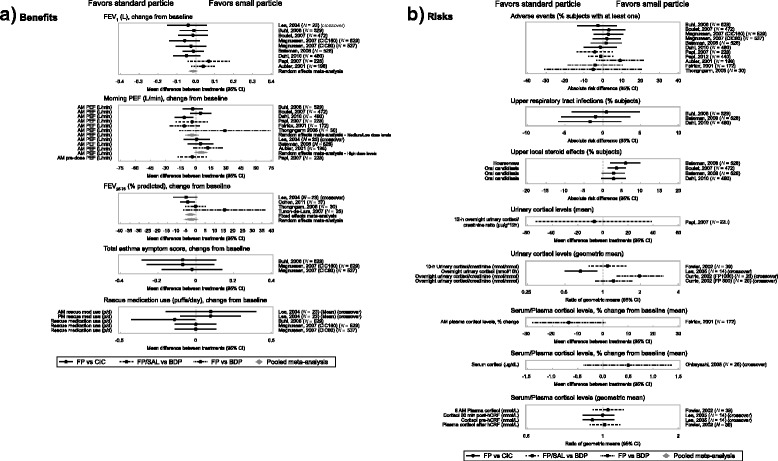
Benefit-risk plot in adolescents and adults by particle size. AM, morning; BDP, beclometasone dipropionate; CI, confidence interval; CIC, ciclesonide; CO, crossover; FEV_1_, forced expiratory volume in 1 s; FEF_25–75%_, % predicted forced expiratory flow between 25% and 75% of forced vital capacity; FP, fluticasone propionate; FP/SAL, fluticasone propionate/salmeterol; hCRF, human corticotropin-releasing factor; PEF, peak expiratory flow; PM, evening

**Fig. 4:**
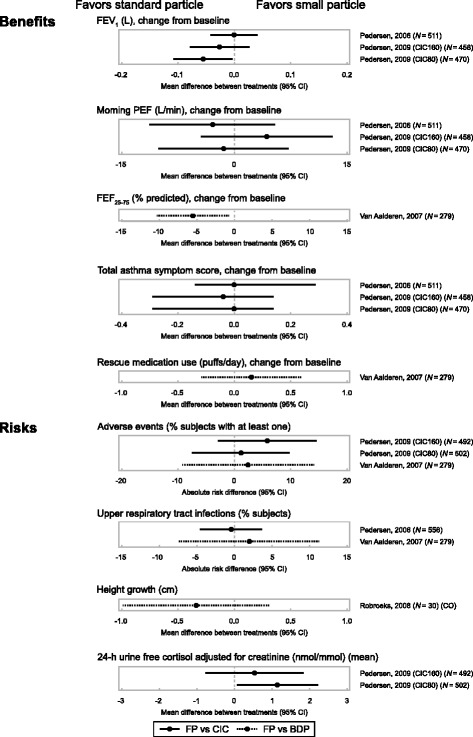
Benefit-risk plot in children by particle size. BDP, beclometasone dipropionate; CIC, ciclesonide; FEF_25–75%,_ % predicted forced expiratory flow between 25% and 75% of forced vital capacity; FEV_1_, forced expiratory volume in 1 s; FP, fluticasone propionate; PEF, peak expiratory flow

The benefit-risk plots included effect estimates from all individual studies included in the analysis of each efficacy and safety endpoint. For adult/adolescent patients (aged ≥12 years), the effect on total asthma symptom score and rescue medication use was only assessed for FP versus CIC. No clinically meaningful differences were noted across the five efficacy endpoints considered (FEV_1_, morning PEF, and FEF_25–75%_, asthma symptoms and rescue medication use) in both adults (Fig. 3) and children (Fig. 4).

No appreciable differences were noted for most safety endpoints (AEs, local steroid effects, upper respiratory tract infections, growth and bone metabolism, and adrenal suppression) in both adults and children (Figs. 3 and 4); most studies though were not designed to test treatment differences for safety endpoints. Patients receiving FP experienced more local steroid effects at the upper airways than those receiving CIC; this was likely due to the fact that CIC is administered as a pro-drug which is only activated in the lower airways. The cortisol levels data were variable, with no clear differentiation between treatments.

##### Publication bias

**Fig. 5:**
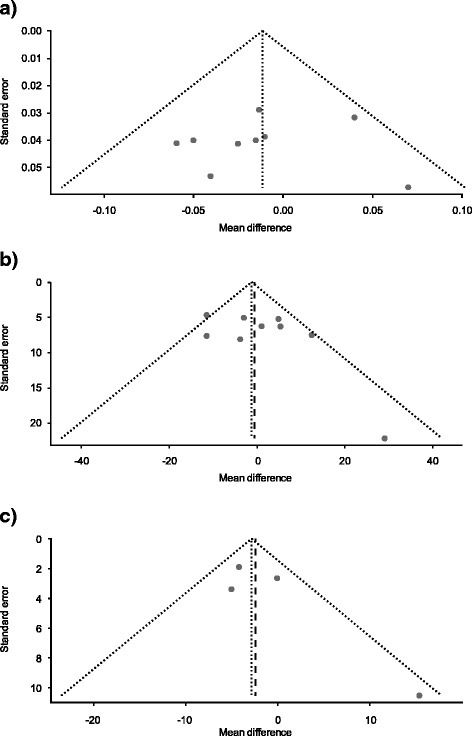
Funnel plots for studies included in the (**a**) FEV_1_, (**b**) morning PEF and (**c**) FEF_25–75%_ endpoints meta-analyses. **a**. Linear regression test of funnel plot asymmetry: t = −0.2342, df = 7, *p*-value = 0.822. **b**. Linear regression test of funnel plot asymmetry: t = 1.332, df = 7, *p*-value = 0.225. **c**. Linear regression test of funnel plot asymmetry t = 1.547, df = 6, *p*-value = 0.262. FEF_25–75%,_ % predicted forced expiratory flow between 25% and 75% of forced vital capacity; FEV_1_, forced expiratory volume in 1 s; PEF, peak expiratory flow

Based on the funnel plots and the test of asymmetry (*p*-values ranging from 0.225–0.822) (Fig. 5), the FEV_1_, morning PEF and PEF_25–75%_ data did not exhibit asymmetry, which suggests that there is neither publication bias nor a systematic difference between smaller and larger studies (‘small study effects’).

## Discussion

### Summary

In this meta-analysis of studies in adults and adolescents, no significant differences were observed between standard size (FP and FP/SAL) and small size (BDP, CIC and BDP-F) particles ICS for change in FEV_1_, morning PEF or FEF_25–75%_ using the random effects model. Similarly, no significant differences were observed between standard size and small size particles ICS in the subgroup analysis according to dose for morning PEF. However, it was observed that the result of the medium/low dose analysis for morning PEF was slightly in favor of standard particles (i.e. standard size particles ICS demonstrated better efficacy) versus small particles while the converse was true for high/high-medium dose analysis. This observed difference in the subgroup analyses suggests that dose level may be the effect modifier for morning PEF.

FEF_25–75%_ was chosen as an efficacy endpoint as it is a more sensitive indicator for disease in the small airways than FEV_1_ [9], and thus more likely to demonstrate variations in efficacy if the smaller particles were meeting the small airways. However, there is ongoing debate as to the role of FEV_25–75%_ values in assessing asthma control and phenotype-driven treatment [33, 34]. Although there were no significant differences in FEF_25–75%_ for different particle sizes using the random effect model (mostly due to the small number of studies), a statistically significant difference in favor of standard particles was seen when the fixed effects model was used because the heterogeneity tests were not significant. However, this statistical difference was not clinically significant.

In terms of specific treatment comparisons, little or no differences in (FEV_1_ and PEF) were reported for FP versus CIC, FP/SAL versus BDP-F, and FP versus BDP. There were no significant differences in FEF_25–75%_ for FP versus CIC; however, evidence of increased efficacy in FEF_25–75%_ was demonstrated in one trial with FP versus BDP [18] while the opposite was shown in another [16]. The results of this study also showed no significant differences in asthma symptoms and use of rescue medications between small and standard size ICS. However, these results should be treated with caution as these parameters were only evaluated between FP and CIC and not for FP versus BDP.

For the safety endpoints considered, no appreciable differences were observed for most endpoints though it should be noted that the majority of studies were not designed to test treatment differences for safety endpoints. Adult/adolescent patients experienced more local steroid effects with standard particles than small particles. The observation that the local steroid effects at the upper airways favored small particles was likely due to the fact that CIC is administered as a pro-drug which is only activated in the lower airways. Overall, the cortisol data were variable with no clear differentiations between particle sizes.

### Particle size, lung deposition, and clinical outcomes

Although there is a relationship between smaller particle size and increased delivery to the distal lung [35, 36], the current study demonstrated that increased deposition in the distal lung does not appear to translate into improved clinical outcomes for patients with asthma. These results are unsurprising for a number of reasons, not least because an increased proportion of the inhaled dose is likely to deposit in the distal respiratory compartment beyond the conducting airways. Based on current understanding, the effect of using an aerosol with smaller particle size or a ‘finer’ aerosol is both to increase the dose reaching the lungs by reducing the oropharyngeal deposition and to increase the proportion of that dose depositing distally. While simplistically this increased distal deposition might be considered to result in increased deposition in the ‘small airways’, it is important to recognize the increased deposition in fact occurs in the respiratory compartment beyond the conducting airways.

In considering the impact of ICS with different particle sizes it is important to understand the factors influencing the pattern of deposition of an aerosol in the complex 3D structure formed by the conducting airways (large and small) and pulmonary/alveolar compartments of the lung. Current mathematical modeling methods combined with 3D imaging suggest that the vast majority of inhaled aerosol (>90%) inhaled during tidal breathing is delivered to the pulmonary/alveolar compartment beyond the conducting airways, and this will increase with the modified inspiratory breath when using a pMDI (with or without a holding chamber) [37, 38]. Hence for a given dose administered to the conducting airways (large, medium, and small), the dose delivered more distally will be relatively greater with the ‘finer’ aerosol. With such a small proportion of the aerosol depositing in the conducting airways it is unlikely that there will be a major change in the concentration of aerosol at the epithelial surface of the conducting airways. This is particularly true of the small conducting airways which have a much greater relative surface area than the more central airways.

Another point of consideration is the conjecture that ‘finer’ aerosols will penetrate more effectively in the face of airways narrowing. However, any perceived advantage of finer aerosols in accessing the blocked/narrowed airways would be transient in nature as it has been shown that the blockage is resolved very rapidly in the vast majority of patients with asthma when they commence ICS treatment [39].

Based on the results of this study and discussions above, it is evident that the key issue should be the evaluation of the ‘therapeutic index’ of different drug-device combinations rather than a comparison of aerosol particle sizes for controlling asthma. Unfortunately, there is no robust method for assessing this. As previously mentioned, apart from particle size, drug deposition within the lung is dependent on other factors such as inhaler device and inhalation technique, which varies between patients [36, 40, 41]. Thus, these factors make it impossible to know what lung doses will be achieved when an individual patient uses a particular drug-device combination, even under controlled conditions [42]. Consequently current guidelines advocate titration of ICS dosages against symptoms and spirometric data [2]. Using the lowest effective dose ensures maximum efficacy and minimizes the risk of side effects. The guidelines do not distinguish between corticosteroids formulations and this approach is supported by this systematic review.

A number of observational studies and historical matched cohort analyses have recently been published comparing the outcomes and cost of treatment with small versus standard size particle ICS in patients with asthma [43–46]. In general, the studies found that asthma treatment outcomes were similar or better with small size particle ICS (BDP) compared with standard size particle ICS (FP). However, such studies are usually confounded by variables that are not present in RCTs. For example, observational studies often rely on prescription data (which does not necessarily translate to actual dosage taken) or are not able to quantify past exposure of some drugs. Furthermore, control for asthma severity in these studies was often indirect via rescue medication and hospitalizations. Another factor that might confound the results of these studies is the lack of patient randomization.

Most of the individual clinical studies included in this review may not have been powered to detect clinically meaningful differences but statistically significant differences. This meta-analysis offered the opportunity to increase the sample size and power to calculate pooled estimates for treatment differences. Despite this increased power to detect statistical differences, the relevance and clinical meaningfulness of these results must be determined beyond the results showing statistical significance.

A potential limitation of literature reviews/meta-analyses pertains to publication bias. Searches of databases such as PubMed or EMBASE yield long lists of studies that have been published. Such searches are unlikely to yield a representative sample because studies that show a ‘positive’ result are more likely to be published than those that do not. However, based on the funnel plots and the test of asymmetry, the FEV_1_, morning PEF and FEF_25–75%_ data did not exhibit asymmetry, which suggests that publication bias is not likely to be a limiting factor in this study.

Another potential limitation is that the present study did not explore whether other parameters of inflammation such as fractional exhaled nitric oxide [47] were differentially affected by particle size. Similarly, endpoints such as asthma exacerbations, which together with lung function and asthma symptoms indicate sub-optimal asthma control [1], was also not assessed in the present study.

## Conclusion

In summary, the results of this systematic review do not support the suggestion that smaller size particle ICS are intrinsically more ‘effective’ than larger standard size particle ICS on the endpoints of lung function, asthma symptoms and rescue medication use. Markers of inflammation and asthma exacerbation were not assessed in this meta-analysis and so the ability of small particle treatments to differentially affect these outcomes were not possible to ascertain. No study to date has clearly addressed the key issue of the relative therapeutic index of the different drug-delivery combinations though there are robust data that regular (>80% of doses) use of these treatments at licenced doses is effective and well tolerated.

## Abbreviations

AE: Adverse event
AM: Morning
BDP: Beclometasone dipropionate
BDP-F: Beclometasone dipropionate-formoterol
CFC: Chlorofluorocarbon
CI: Confidence interval
CIC: Ciclesonide
CL: Clearance
CO: crossover
CV-VC: Closing volume/vital capacity
DPI: Dry powder inhaler
dx: Diagnosis
ER: Emergency room
FEF: Forced expiratory flow
FEV_1_: Forced expiratory volume in 1 s
FP: Fluticasone propionate
FP/SAL: Fluticasone propionate-salmeterol combination
FVC: Forced vital capacity
hCRF: human corticotropin-releasing factor
HFA: Hydrofluoroalkane
HFA-pMDI: Hydrofluoroalkane-pressurized metered dose inhaler
ICS: Inhaled corticosteroids
ITT: Intent-to-treat
MMAD: Mass median aerodynamic diameter
NO: Nitric oxide
NR: Not reached
PEF: Peak expiratory flow
PEFR: Peak expiratory flow rate
PM: Evening
PMDI: Pressurized metered dose inhalers
PP: Per protocol
ppb: Part per billion
RCT: Randomized controlled trial
RV: Residual volume
SABA: Short-acting beta agonists
SE: Standard error
SVC: Slow vital capacity
TGV: Thoracic gas volume
TLC: Total lung capacity
Tx: Treatment
