# Correction to: Effect of inhaled corticosteroid particle size on asthma efficacy and safety outcomes: a systematic literature review and meta-analysis

**DOI:** 10.1186/s12890-017-0507-2

**Published:** 2017-12-05

**Authors:** Céline El Baou, Rachael L. DiSantostefano, Rafael Alfonso-Cristancho, Elizabeth A. Suarez, David Stempel, Mark L. Everard, Neil Barnes

**Affiliations:** 1 GSK, Middlesex, Stockley Park, Uxbridge UK; 2 GSK, Research Triangle Park, NC USA; 3 GSK, Upper Providence, PA USA; 4 University of Washington, School of Medicine, Seattle, WA USA; 5 New England Research Institutes, Watertown, MA USA; 6 The University of Western Australia, Crawley, WA 6009 Australia; 7 GSK, Brentford, UK; 8 William Harvey Research Institute, Barts and The London School of Medicine and Dentistry, London, UK; 9 Current affiliation: Janssen Pharmaceuticals, Titusville, NJ USA; 10 Current affiliation: University of North Carolina at Chapel Hill, Chapel Hill, NC USA; 11 PHASTAR, Chiswick, London UK

## Correction

After publication of this work [1] it was noticed that the author name Rachael L. DiSantostefano was not spelt correctly as there was a space in her surname between ‘Di’ and ‘Santostefano’.

The publisher apologises for this error.
